# Repeated inflammatory dural stimulation-induced cephalic allodynia causes alteration of gut microbial composition in rats

**DOI:** 10.1186/s10194-022-01441-9

**Published:** 2022-06-25

**Authors:** Shuai Miao, Wenjing Tang, Heng Li, Bozhi Li, Chunxiao Yang, Wei Xie, Tao Wang, Wenhao Bai, Zihua Gong, Zhao Dong, Shengyuan Yu

**Affiliations:** 1 Department of Neurology, the First Medical Center, Chinese PLA General Hospital, Fuxing Road 28, Haidian District, 100853 Beijing, People’s Republic of China; 2 Medical School of Chinese PLA, 100853 Beijing, People’s Republic of China; 3 Department of Neurology, The First Affiliated Hospital of Shandong First Medical University, Jinan, China; 4 School of Medicine, Nankai University, Tianjin, China

**Keywords:** Migraine, Cephalic allodynia, Gut microbiota, 16 S rRNA gene sequencing, Gut-brain axis

## Abstract

**Background:**

Gut microbial dysbiosis and gut-brain axis dysfunction have been implicated in the pathophysiology of migraine. However, it is unclear whether migraine-related cephalic allodynia could induce the alteration of gut microbial composition.

**Methods:**

A classic migraine rat model was established by repeated dural infusions of inflammatory soup (IS). Periorbital mechanical threshold and nociception-related behaviors were used to evaluate IS-induced cephalic allodynia and the preventive effect of topiramate. The alterations in gut microbial composition and potential metabolic pathways were investigated based on the results of 16 S rRNA gene sequencing. Microbiota-related short-chain fatty acids and tryptophan metabolites were detected and quantified by mass spectrometry analysis.

**Results:**

Repeated dural IS infusions induced cephalic allodynia (decreased mechanical threshold), migraine-like behaviors (increased immobility time and reduced moving distance), and microbial composition alteration, which were ameliorated by the treatment of topiramate. Decreased *Lactobacillus* was the most prominent biomarker genus in the IS-induced alteration of microbial composition. Additionally, IS infusions also enhanced metabolic pathways of the gut microbiota in butanoate, propanoate, and tryptophan, while the increased tryptophan-related metabolites indole-3-acetamide and tryptophol in feces could be the indicators.

**Conclusions:**

Inflammatory dural stimulation-induced cephalic allodynia causes the alterations of gut microbiota profile and microbial metabolic pathways.

## Background

Migraine, a highly prevalent and debilitating disorder in neurology, affects approximately 14% of the adult population worldwide [1]. Although activation of the trigeminovascular system is recognized responsible for the characteristics of headache in migraine, the exact pathogenesis of migraine is not well understood [2]. Migraine attacks are often accompanied by gastrointestinal complaints such as nausea and vomiting. Functional gastrointestinal disorders, including irritable bowel syndrome (IBS) and inflammatory bowel disease (IBD), are common comorbidities of migraine [3, 4]. These gastrointestinal disorders, together with extraintestinal comorbid neurological diseases, are linked to gut microbial dysbiosis [5]. Previous studies have demonstrated that the gut microbiota not only plays a key role in visceral pain, but also participates in the modulation of chronic pain [6, 7]. Alterations of the gut microbiota and dysfunction of the gut-brain axis are involved in the pathophysiology of migraine [8].

The gut-brain axis refers to bidirectional communications between the gut microbiota and brain, integrating gut and central nervous system (CNS) activities. Brain can influence the composition and function of gut microbiota through the autonomic nervous system and neuroendocrine factors [9]. Reversely, neurotransmitters, inflammatory cytokines, and metabolites from the gut microbiota can propagate signals to affect brain function [8]. Previous studies have revealed that some microbiota-derived products, such as short-chain fatty acids (SCFAs) and tryptophan metabolites, are involved in diverse physiological and pathological activities [10, 11]. In addition to regulating homeostasis and inflammation of the gastrointestinal tract, SCFAs have neuroprotective properties and immunomodulatory effects [8]. Kukkar et al. discovered that SCFAs can attenuate neuropathic pain in a rat model [12]. Accumulating evidence has shown that the gut microbiota directly or indirectly influences tryptophan metabolic pathways, including serotonin (5-hydroxytryptamine, 5-HT), kynurenine, and indole-related derivatives [13]. Central 5-HT metabolism has been demonstrated to play pivotal roles in migraine attacks, while the intestinal microbiota can regulate tryptophan availability and indirectly affect the serotoninergic pathway in the CNS [13, 14]. Additionally, the gut microbiota directly participates in the kynurenine-producing pathway, and its downstream product kynurenic acid (KYNA) plays an important role in pain modulation [15].

In our previous study, we have found that the gut microbiota can modulate mechanical allodynia in a nitroglycerin (NTG)-induced migraine model [16]. However, it is not clear whether migraine-related cephalic allodynia could induce alterations of the gut microbial composition in animal models of migraine. A recent study has shown that intraperitoneal injection with NTG can increase cytokine release and provoke intestinal mucosa collapse in mice [17]. Considering the possible effect of NTG on the intestinal microbiota, we used the inflammatory soup (IS) model instead in this study [18]. The IS migraine model was established by repeated dural infusions of IS, mimicking neurogenic inflammation and trigeminal sensitization in the pathogenesis of migraine [2, 18, 19]. We investigated alterations of the microbiota profile and microbial metabolic pathways based on the results of 16 S rRNA gene sequencing. To detect the levels of microbiota-related products, quantitative profiling of SCFAs and tryptophan metabolites was performed by gas chromatography-tandem mass spectrometry (GC–MS/MS) and liquid chromatography-tandem mass spectrometry (LC–MS/MS), respectively.

## Materials and methods

### Animals

Specific pathogen-free (SPF) male Sprague–Dawley rats (200–220 g) were purchased from SiPeiFu Biotechnology Co., Ltd. (Beijing, China). The rats (total *N* = 32) were housed in a room with controlled temperature (22–25 ℃), humidity (40–60%), a 12:12 h light/dark cycle and free access to food and water. The sample size was calculated by G*Power (ver. 3.1.9.7) based on the repeated measures design (power = 0.85) [20]. The experimental procedures were approved by the Institutional Animal Care and Use Committee, Chinese People’s Liberation Army (PLA) General Hospital, following the Regulations for the Administration of Affairs Concerning Experimental Animals.

### Surgical procedure

After one week of habituation, a cannula was implanted into the skull of each rat as previously described [21]. Briefly, a rat was anesthetized intraperitoneally with 50 mg/kg pentobarbital sodium and placed onto a stereotactic frame. A midline incision was made in the head and all soft tissues were bluntly dissected to expose the bregma. A burr hole, 1.5 mm lateral to the midline and 1.5 mm posterior to bregma, was drilled to expose the dura mater adjacent to the superior sagittal sinus. A stainless steel inner cannula (O.D. 0.64 mm, M3.5), with a matched cap (O.D. 0.40 mm, M3.5) was implanted into the hole and affixed to the skull with tissue adhesive (3 M Vetbond). Dental cements were used to cover the foundation of the cannula for further fixation and the incision was sutured carefully, leaving the plastic cap outside the skin. After cannula surgery, the rats were housed separately for the subsequent experiments.

### Drug delivery and IS infusion

**Fig. 1:**
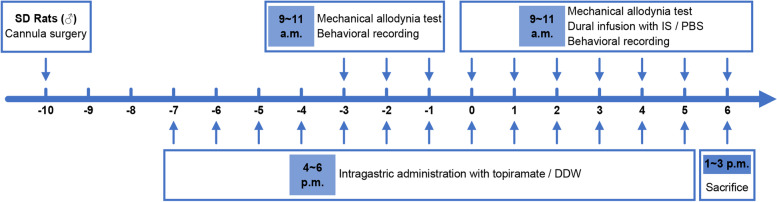
Flow chart of the experimental outline

According to the effective daily doses of 100 mg in chronic migraineurs, the rats received an equivalent dosage of topiramate (10 mg/Kg/day) [22, 23]. A topiramate solution or an equal amount of double distilled water (DDW) was intragastrically administered once daily for a total of 13 consecutive times. The IS contained bradykinin (2 mM), histamine (2 mM), 5-HT (2 mM), prostaglandin E2 (0.2 mM), and phosphate buffered saline (PBS) as a vehicle for solubilization. In awake rats, dural infusions of 10 µL IS or PBS were performed 5 min after the facial mechanical allodynia test, using a microsyringe once daily for a total of 7 consecutive times. Thirty-two rats were randomly divided into 4 groups (*n* = 8): (1) VEH group (DDW administrations, PBS infusions); (2) IS group (DDW administrations, IS infusions); (3) TO-VEH group (topiramate administrations, PBS infusions); (4) TO-IS group (topiramate administrations, IS infusions). As shown in the flow chart of experimental outline (Fig. 1), intragastric administrations and dural infusions were provided continuously from day − 7 and day 0, respectively.

### Facial mechanical allodynia test

Before dural infusion, facial mechanical allodynia of rats was evaluated by periorbital mechanical threshold using von Frey filaments (Aesthesio, Ugo Basile, Italy), as previously described [19, 24]. Each rat was habituated in a plexiglass box for 10 min before measurement. The mechanical detection threshold was measured by applying von Frey filaments to the inner canthus region of rats. The filaments were applied in sequential increasing order with manufacturer-calibrated forced values (0.07, 0.16, 0.4, 0.6, 1, 1.4, 2, 4, 6 g). A positive response was considered when the rat quickly retracted its head away, stroked its face with the ipsilateral forepaw, or exhibited wet-dog shakes. After a positive response, a weaker stimulus was presented. The threshold was defined as a positive response to three of five trials of the monofilament. The von Frey tests were performed by an investigator blind to the treatment groups.

### Behavioral recording and analysis

From day − 3 to day 5, behavioral testing was performed every other day for a total of 5 times. After mechanical allodynia test (day − 3 and day − 1) or dural infusion (day 1, day 3 and day 5), the rat was immediately placed into a black box for a 15-min behavioral recording. An overhead camera monitored the rat’s movement and position, and the behavioral data were collected by CinePlex Studio V3 (Plexon Inc., TX, USA). The moving distance was automatically obtained with a speed threshold of 0.1 cm/s. Immobility time was detected by the Freezing Detector Module of CinePlex Editor V3 (Plexon Inc., TX, USA). After manually analyzing the automatically generated video fragments, we calculated the exact immobility time. The behavioral data were analyzed by an investigator blind to the treatment groups.

### 16 S rRNA gene sequencing

On day 6, all the rats were sacrificed 4 hours after the final IS application. Stool samples were immediately collected from colons into a sterile tube and then stored at -80 ℃. Bacterial genomic DNA was extracted from stool samples using a QIAamp PowerFecal Kit (Qiagen, Hilden, Germany) following the manufacturer’s manual. The V3-4 hypervariable region of the bacterial 16S rRNA gene was amplified with the primers 338F (5’-ACTCCTACGGGAGGCAGCAG-3’) and 806R (5’-GGACTACNNGGGTATCTAAT-3’). Polymerase chain reaction (PCR) was carried out on a Mastercycler Gradient (Eppendorf, Germany), and deep sequencing was performed on an Illumina MiSeq platform. After removing low-quality sequences from raw data, qualified reads were clustered into operational taxonomic units (OTU) at a similarity level of 97% using the Uparse algorithm of Vsearch (ver 2.7.1). The OTUs were assigned to taxa based on the Silva 138 bacterial database for further analysis by Qiime (ver 1.8.0).

### Gas chromatography-tandem mass spectrometry

Levels of SCFAs were detected by GC–MS/MS in both feces and sera, an efficient and sensitive assay to analyze volatile compounds. Fecal samples were obtained as described above and blood samples were collected using the abdominal aortic method before transcardial perfusion of the rats. The serous samples were obtained after centrifugation (4200 rpm, 10 min) and stored at -80 °C. All the samples were prepared following standard procedures, and analyzed in multiple reaction monitoring (MRM) mode on an Agilent 7890B gas chromatograph coupled to a 7000D mass spectrometer system (Agilent, CA). The MRM parameters for each targeted analyte were optimized by flow injection analysis. Agilent MassHunter (B.08.00, Agilent Technologies) was used for data acquisition and processing.

### Liquid chromatography-tandem mass spectrometry

Levels of tryptophan and its metabolites were detected by LC–MS/MS in both feces and sera. Fecal and serous samples were obtained as described above. The prepared samples were analyzed using an LC–ESI–MS/MS system, including ultra-performance liquid chromatography (UPLC, ExionLC AD) and tandem mass spectrometry (MS/MS, QTRAP 6500+). Quantitative analysis of tryptophan metabolites was performed by MRM. Analyst (ver 1.6.3, Sciex) and Multiquant (ver 3.0.3, Sciex) were used for data acquisition and analyte quantification, respectively.

### Statistical analysis

Principal coordinates analysis (PCoA) at the OTU level was performed to assess *β*-diversity, while community dissimilarities were detected by permutational multivariate analysis of variance (PERMANOVA, specifically the Adonis test). Analysis of similarities (ANOSIM) was used to identify between- and within-group similarities. The biomarker taxa responsible for discrimination were detected by the linear discriminant analysis (LDA) effect size (LEfSe, https://huttenhower.sph.harvard.edu/galaxy/) [25]. Random forest (RF) analysis was performed by the R package “randomForest”, and the mean decrease accuracy (MDA) score was used to determine the importance ranking of key taxa for classification [26]. Based on the Kyoto Encyclopedia of Genes and Genomes (KEGG) database, microbial functional pathways were predicted by the R package “Tax4Fun2”, and the result was presented with an extended error bar plot using STAMP (ver 2.1.3) [27, 28].

To compare statistical differences between two groups, we used a Student’s t-test, a Welch’s t-test (equal variances not assumed) or a Wilcoxon rank-sum test (non-parametric data). To control the false discovery rate (FDR) for multiple testing, the q-value (corrected *p*-value) was calculated using the Benjamini–Hochberg method. Repeated-measures data were analyzed by repeated-measures analysis of variance (ANOVA), followed by Bonferroni or Fisher’s least significant difference (LSD) for post hoc tests. SPSS 22.0 and R 4.1.0 were used for the data analysis described above, and *P* < 0.05 was considered significant.

## Results

### Periorbital mechanical threshold

**Fig. 2:**
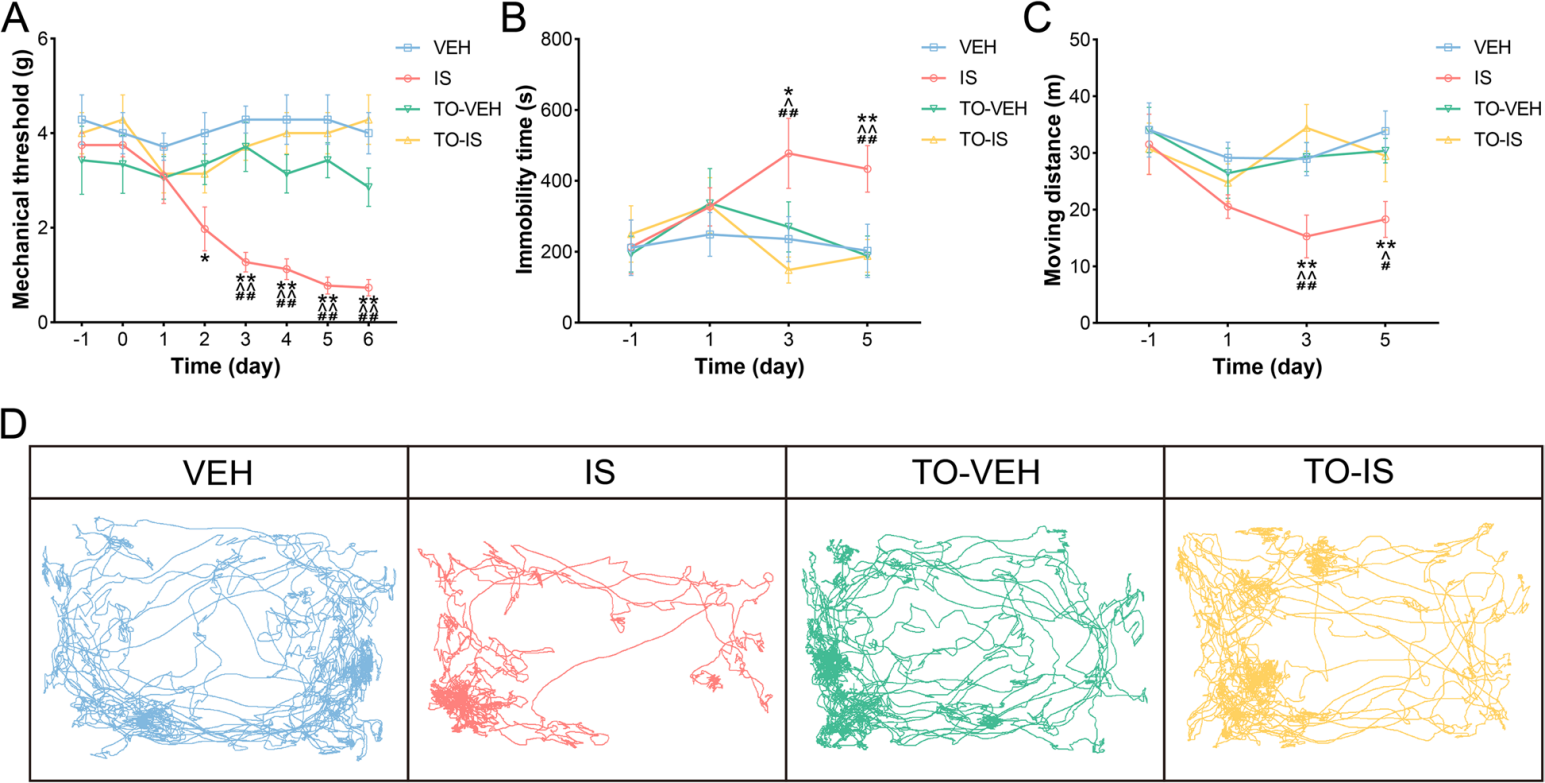
Effects of repeated dural IS infusions on behavioral characteristics. **A** Comparisons of periorbital mechanical threshold among groups. **B** Comparisons of immobility time among groups. **C** Comparisons of the moving distance among groups. **D** Representative trajectory of each group on day 5. Data are presented as the mean ± SEM (*n* = 8; *P < 0.05, **P < 0.01, compared with the VEH group; ^*P* < 0.05, ^^*P* < 0.01, compared with the TO-VEH group; ^#^*P* < 0.05, ^##^*P* < 0.01, compared with the TO-IS group)

Basal mechanical thresholds (day − 1 and day 0) showed no significant differences among the four groups (Fig. 2 A). After infusing the IS twice, we found that the threshold on day 2 decreased significantly in the IS group compared with the VEH group (*P* < 0.05). From day 3 to day 6, the thresholds in the IS group were significantly different from those in the other three groups (IS vs. VEH, *P* < 0.01; IS vs. TO-VEH, *P* < 0.01; IS vs. TO-IS, *P* < 0.01). These results showed that repeated dural IS infusions induced cephalic allodynia in rats, which could be ameliorated by intragastrical preconditioning with topiramate.

### Immobility time and moving distance

As shown in Fig. 2B, in comparison with the other three groups, significantly increased immobility time in the IS group was detected on day 3 (IS vs. VEH, *P* < 0.05; IS vs. TO-VEH, *P* < 0.05; IS vs. TO-IS, *P* < 0.01) and day 5 (IS vs. VEH, *P* < 0.01; IS vs. TO-VEH, *P* < 0.01; IS vs. TO-IS, *P* < 0.01). Similarly, statistically decreased moving distance in the IS group was found on day 3 (IS vs. VEH, *P* < 0.01; IS vs. TO-VEH, *P* < 0.01; IS vs. TO-IS, *P* < 0.01; Fig. 2 C) and day 5 (IS vs. VEH, *P* < 0.01; IS vs. TO-VEH, *P* < 0.05; IS vs. TO-IS, *P* < 0.05) compared with the other three groups. Representative trajectory of each group on day 5 was presented in Fig. 2D. The above demonstrated that repeated dural IS infusions induced migraine-like behaviors in rats, including increased immobility time and decreased movement distance, which could be ameliorated by intragastrical preconditioning with topiramate.

### Beta-diversity analysis

**Fig. 3:**
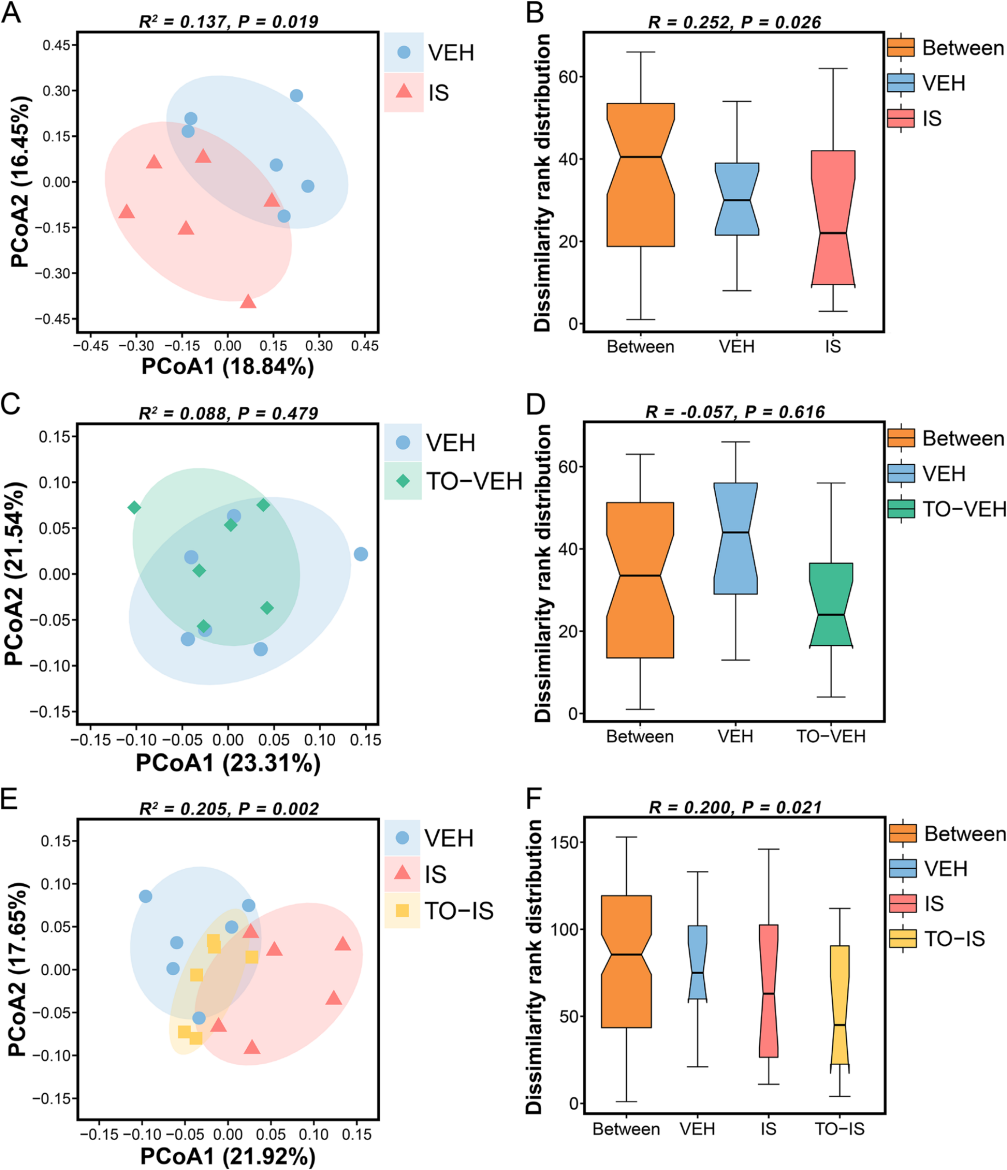
Beta-diversity of gut microbiota at the OTU level. **A** PCoA plot between the VEH and IS groups. **B** ANOSIM plot between the VEH and IS groups. **C** PCoA plot between the VEH and TO-VEH groups. **D** ANOSIM plot between the VEH and TO-VEH groups. **E** PCoA plot among the VEH, IS and TO-IS groups. **F** ANOSIM plot among the VEH, IS and TO-IS groups. In PCoA plots, each symbol represents the data of an individual sample and an R-square value (range [0, 1]) shows a given grouping factor to determine the variation of distances (Adonis test, Euclidean distance, *n* = 6). In ANOSIM plots, the *x*-axis represents the different groups and the *y*-axis represents the distance rank of samples (ANOSIM test, Bray–Curtis distance, *n* = 6). An R-value (range [-1, 1]) greater than zero indicates that intergroup dissimilarities are greater than intragroup dissimilarities, while an R-value less than zero means that there are no intergroup differences

A significant difference was detected in OTU compositions between the VEH and IS groups (*R* ^*2*^_*Adonis*_ = 0.137, *P*_*Adonis*_ = 0.019; *R*_*ANOSIM*_ = 0.252, *P*_*ANOSIM*_ = 0.026; Fig. 3 A and B). However, no statistical difference was found in OTU compositions between the VEH and TO-VEH groups (*R*^*2*^_*Adonis*_ = 0.088, *P*_*Adonis*_ = 0.479; *R*_*ANOSIM*_ = -0.057, *P*_*ANOSIM*_ = 0.618; Fig. 3 C and D). In addition, there were significant differences in OTU compositions among the VEH, IS and TO-IS groups (*R*^*2*^_*Adonis*_ = 0.205, *P*_*Adonis*_ = 0.002; *R*_*ANOSIM*_ = 0.200, *P*_*ANOSIM*_ = 0.021; Fig. 3E and F). Results of post hoc tests showed that significant difference was detected between the IS and TO-IS groups (*R*^*2*^_*Adonis*_ = 0.170, *P*_*Adonis*_ = 0.024), but no statistical difference was found between the VEH and TO-IS groups (*R*^*2*^_*Adonis*_ = 0.118, *P*_*Adonis*_ = 0.110). These results revealed the following: (1) repeated dural IS infusions caused microbial composition alteration in rats; (2) treatment with topiramate did not influence the gut microbiota composition in rats; (3) intragastrical preconditioning with topiramate might prevent IS-induced microbial composition alteration in rats.

### Microbial distributions at the phylum and family levels

**Fig. 4:**
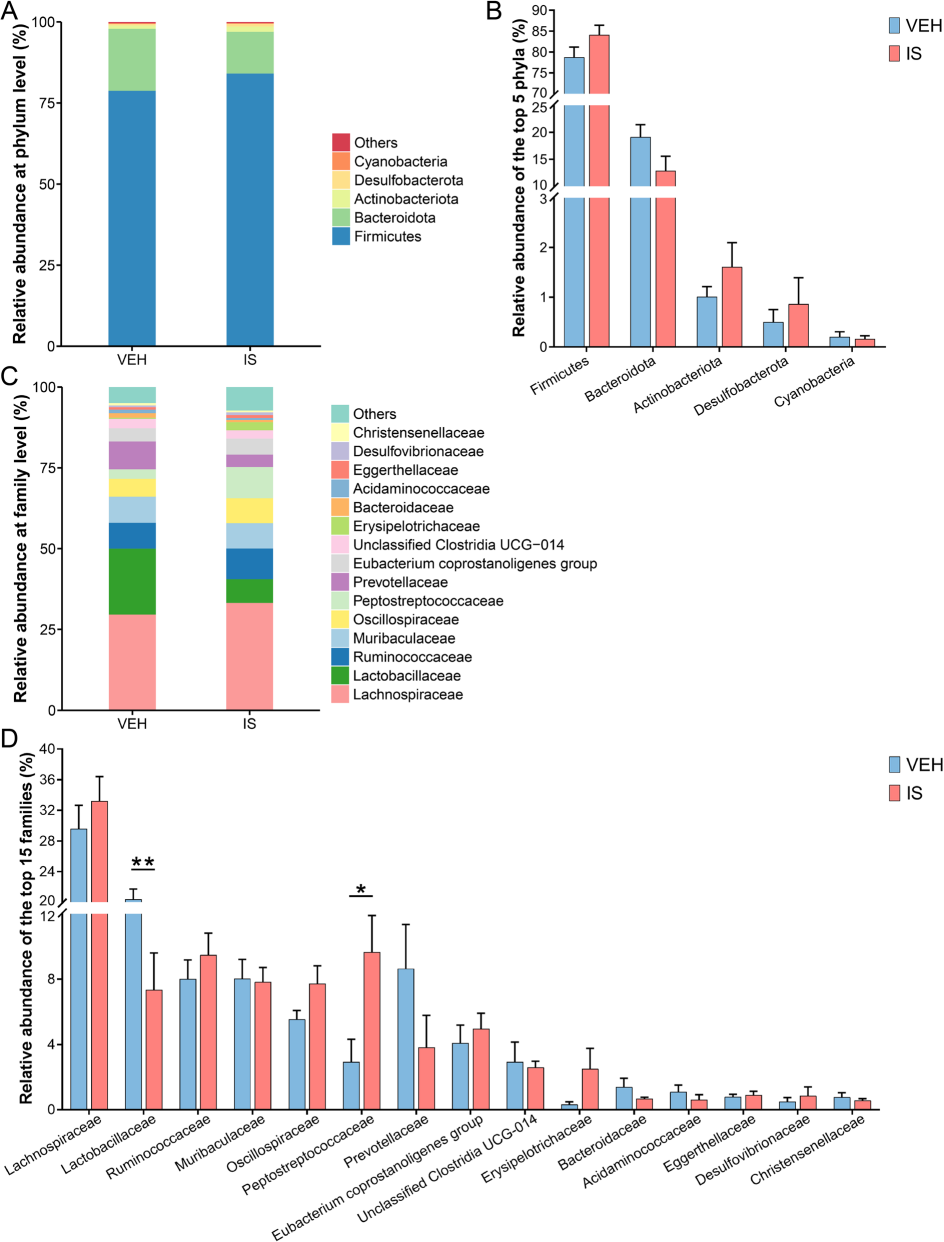
Microbial distributions at the phylum and family levels. **A** Community bar plot at the phylum level. **B** Relative abundance of the top 5 most abundant phyla. **C** Community bar plot at the family level. **D** Relative abundance of the top 15 most abundant families. Data are presented as the mean ± SEM (*n* = 6; *P < 0.05, **P < 0.05)

At the phylum and family levels, microbial distributions of the most abundant taxa were analyzed between the VEH and IS groups. The community bar plot at the phylum and family levels showed the distributions of the top 5 most abundant phyla and the top 15 most abundant families, respectively (Fig. 4 A and C). At the phylum level, no significantly different bacteria were found (*P* > 0.05; Fig. 4B). However, at the family level, both *Lactobacillaceae* and *Peptostreptococcaceae* showed significantly different (*P* < 0.05; Fig. 4D). These results showed that repeated dual IS infusions induced altered abundances at the family level, including increased *Peptostreptococcaceae* and reduced *Lactobacillaceae*.

### Biomarker taxa at the genus level

**Fig. 5:**
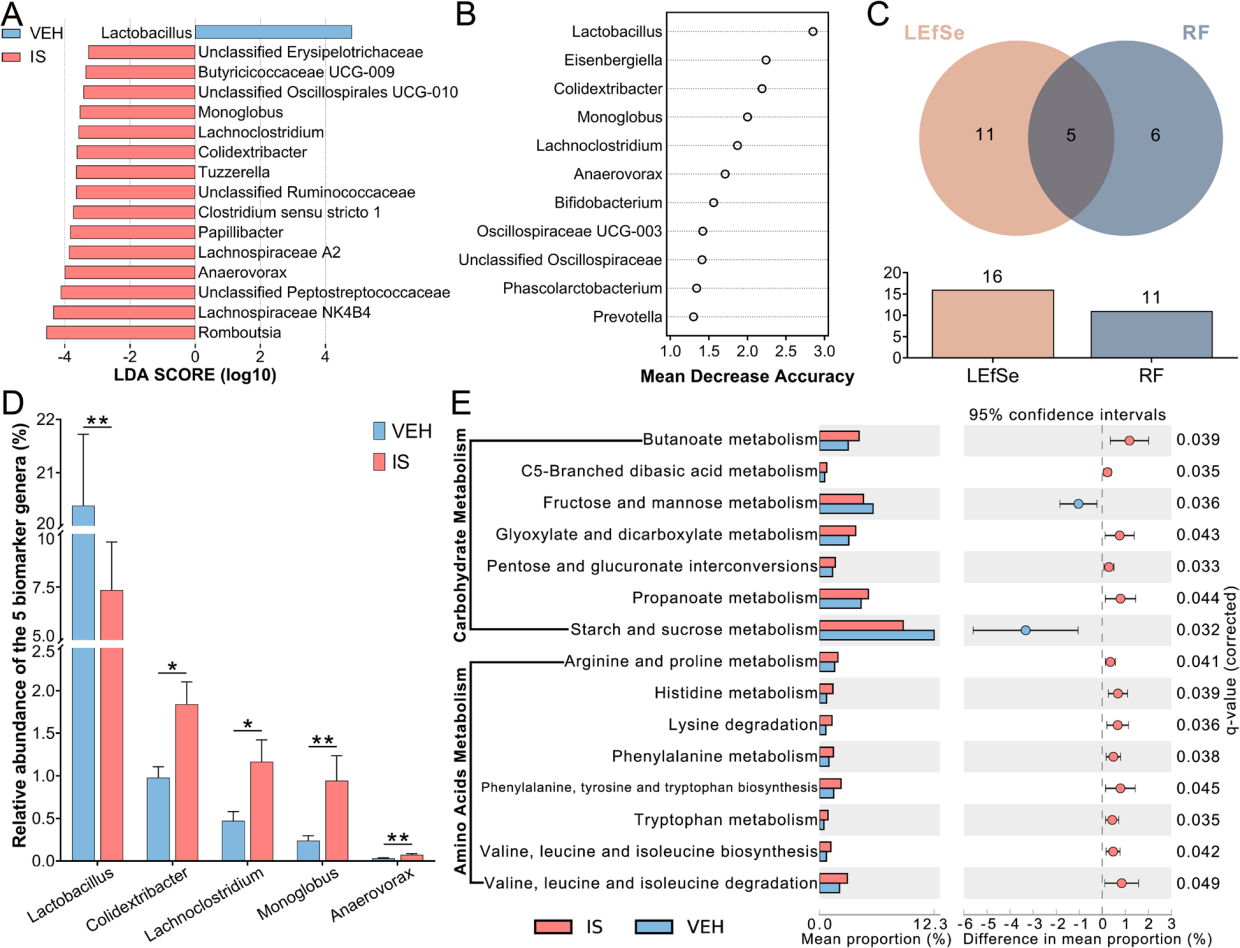
Analyses of the biomarker genus and differential pathways of carbohydrate and amino acid metabolism. **A** LEfSe analysis at the genus level. The bars represent the effect size (LDA score) of significantly discriminative genera. **B** Random forest analysis at the genus level. The y-axis displays the key genera ranked by their relative importance (MDA score) for classification. **C** Common biomarker genera in the LEfSe and RF analyses. **D** Relative abundances of the 5 biomarker genera. Data are presented as the mean ± SEM (*n* = 6; *P < 0.05, **P < 0.01) **E** Comparison of Tax4Fun2-predicted microbial functional pathways of carbohydrate and amino acid metabolism. The extended error bars plot shows differential metabolic pathways (Welch’s t-test, Benjamini–Hochberg FDR)

To detect the biomarker genera, LEfSe analysis and RF analysis were performed between the IS and VEH groups. Based on the logarithmic LDA score of 3.0 as the cutoff, the LEfSe analysis revealed 16 differentially abundant genera (Fig. 5A). In addition, as shown in the plot from the RF analysis (MDA score > 1.0, Fig. 5B), 11 key genera for classification were found. Given the results of these two analyses, we identified five common genera as biomarker taxa, including *Lactobacillus*, *Colidextribacter*, *Lachnoclostridium*, *Monoglobus* and *Anaerovorax* (Fig. 5 C). Compared with the VEH group, the relative abundances of *Colidextribacter*, *Lachnoclostridium*, *Monoglobus* and *Anaerovorax* were significantly increased in the IS group, while that of *Lactobacillus* was significantly decreased (Fig. 5D). Based on the highest LDA score as well as MDA score for *Lactobacillus*, we considered that reduced *Lactobacillus* was the most prominent biomarker genus in the IS-induced alteration of microbiota composition.

### Microbial functional predictions

The extended error bar plot showed differential pathways predicted by Tax4Fun2 from level 3 KEGG orthologs, including carbohydrate and amino acid metabolisms (Welch’s t-test, q-value < 0.05; Fig. 5E). In comparison to the VEH group, most metabolic pathways were enhanced in the IS group, except for fructose and mannose, starch and sucrose metabolisms. Among these pathways, we were interested in SCFAs and tryptophan metabolisms, which were linked to pain modulation and migraine pathophysiology. The above indicated that the butanoate, propanoate, and tryptophan metabolic pathways were enhanced in the IS-induced alteration of microbial metabolism.

### Levels of SCFAs in both feces and sera

To find the altered levels of SCFAs, 7 kinds of SCFAs were detected by GC–MS/MS (Tables 1 and 2). However, both fecal and serous SCFAs showed no significant difference between the IS and VEH groups.

**Table 1:** Levels of SCFAs in feces (mean ± SD)

	IS group	VEH group	*p*-value
AA (mg/g)	0.48 ± 0.16	0.45 ± 0.15	0.74
PA (mg/g)	0.21 ± 0.05	0.29 ± 0.14	0.23
IBA (ng/g)	22.7 ± 10.1	17.7 ± 10.9	0.42
BA (mg/g)	0.15 ± 0.03	0.25 ± 0.19	0.26
IVA (ng/g)	15.3 ± 4.64	11.8 ± 4.59	0.22
VA (ng/g)	28.3 ± 10.9	28.3 ± 20.4	0.99
CA (ng/g)	54.2 ± 75.6	39.3 ± 55.6	0.71

*AA* acetic acid, *PA* propionic acid, *IBA* isobutyric acid, *BA* butyric acid, *IVA* isovaleric acid, *VA* valeric acid, *CA* caproic acid

**Table 2:** Levels of SCFAs in sera (mean ± SD)

	IS group	VEH group	*p*-value
AA (µg/mL)	6.04 ± 8.11	4.73 ± 6.42	0.59
PA (ng/mL)	33.3 ± 17.7	34.0 ± 29.0	0.96
IBA (ng/mL)	15.9 ± 11.6	14.0 ± 8.05	0.94
BA (µg/mL)	0.08 ± 0.02	0.10 ± 0.03	0.09
IVA (ng/mL)	24.6 ± 17.5	30.5 ± 11.6	0.39
VA (ng/mL)	1.95 ± 1.32	6.46 ± 8.07	0.21
CA (ng/mL)	40.3 ± 19.0	48.2 ± 37.6	0.66

### Levels of tryptophan metabolites in both feces and sera

To find the altered products of tryptophan metabolism, 16 kinds of tryptophan metabolites were detected by LC–MS/MS (Tables 3 and 4). Serous tryptophan metabolites showed no statistical differences between the IS and VEH groups. However, the levels of indole-3-acetamide (IAM) and tryptophol (indoleethanol, IEt) in feces were significantly increased in the IS group compared with the VEH group (*P* < 0.05). These results showed that increased IAM and IEt in feces could be the indicators in the IS-induced alteration of microbial metabolism.

**Table 3:** Levels of tryptophan metabolite in feces (mean ± SD)

	IS group	VEH group	*p*-value
L-TRP (µg/g)	5.83 ± 2.36	7.77 ± 3.27	0.27
5-HT (µg/g)	1.10 ± 0.79	0.62 ± 0.33	0.21
NAS (µg/g)	0.13 ± 0.13	0.10 ± 0.03	0.55
5-HIAA (µg/g)	0.32 ± 0.20	0.32 ± 0.14	0.99
L-KYN (ng/g)	34.1 ± 12.5	40.3 ± 19.5	0.53
KYNA (µg/g)	0.50 ± 0.71	0.24 ± 0.21	0.09
XA (µg/g)	0.06 ± 0.03	0.15 ± 0.10	0.09
PiA (µg/g)	3.82 ± 2.72	4.36 ± 3.21	0.76
IAM (ng/g)	32.5 ± 8.83	12.8 ± 2.07	0.02*
IAA (µg/g)	0.81 ± 0.48	1.19 ± 0.98	0.42
IAld (µg/g)	0.29 ± 0.12	0.38 ± 0.16	0.33
TRM (µg/g)	0.81 ± 0.43	1.16 ± 0.38	0.17
IEt (µg/g)	0.12 ± 0.05	0.06 ± 0.03	0.03*
ILA (ng/g)	24.2 ± 17.8	39.5 ± 53.2	0.70
IA (ng/g)	32.4 ± 15.1	29.9 ± 8.63	0.73
IPA (µg/g)	0.54 ± 0.37	0.60 ± 0.28	0.75

*L-TRP* L-tryptophan, *5-HT* serotonin, *NAS* N-acetylserotonin, *5-HIAA* 5-hydroxyindoleacetic acid, *L-KYN* L-kynurenine, *KYNA* kynurenic acid, *XA* xanthurenic acid, *PiA* picolinic acid, *IAM* indole-3-acetamide, *IAA β*-indole-3-acetic acid, *IAld* indole-3-aldehyde, *TRM* tryptamine, *IEt* tryptophol (indoleethanol), *ILA* indole-3-lactic acid, *IA* indole-3-*β*-acrylic acid, *IPA* indole-3-propionic acid

**Table 4:** Levels of tryptophan metabolites in sera (mean ± SD)

	IS group	VEH group	*p*-value
L-TRP (µg/mL)	8.89 ± 1.45	9.09 ± 2.13	0.85
5-HT (µg/mL)	2.01 ± 0.32	1.79 ± 0.19	0.19
NAS (ng/mL)	1.05 ± 0.42	0.89 ± 0.58	0.59
5-HIAA (ng/mL)	16.8 ± 5.21	13.2 ± 7.59	0.36
L-KYN (µg/mL)	0.50 ± 0.15	0.49 ± 0.14	0.96
KYNA (ng/mL)	9.86 ± 11.1	15.3 ± 14.8	1.00
XA (ng/mL)	26.1 ± 4.64	29.0 ± 12.6	0.62
IAA (µg/mL)	0.10 ± 0.03	0.11 ± 0.04	0.58
IAld (ng/mL)	1.08 ± 0.70	1.28 ± 0.57	0.39
IEt (ng/mL)	1.18 ± 1.67	1.10 ± 0.95	0.45
ILA (ng/mL)	29.5 ± 5.12	31.9 ± 7.71	0.54
IA (ng/mL)	30.6 ± 19.4	38.5 ± 13.9	0.44
IPA (µg/mL)	0.42 ± 0.26	0.82 ± 0.56	0.15

Levels of PiA, IAM and TRM could not be detected in sera

## Discussion

The present study firstly investigated whether migraine-related cephalic allodynia could induce microbial composition alteration in a rat model. Our results showed that repeated dural IS infusions induced cephalic allodynia, nociception-related behaviors and microbiota community alterations in rats, which could be ameliorated by preconditioning with topiramate. The biomarker genera and the indicator metabolites were also detected in the altered microbiota profile and microbial metabolism.

Migraine is a condition characterized by a moderate to severe throbbing headache, reduction of routine physical activity and lack of movement. Previous studies have shown that activation of the trigeminovascular system and neurogenic inflammation play pivotal roles in the pathophysiology of migraine [2]. Infusing IS to the dura mater could induce meningeal nociception and activate the trigeminovascular pathway, mimicking onset of headache in migraine [19]. Melo-Carrillo et al. reported decreased exploratory activity and increased resting behavior in IS-induced migraine rats [29]. Consistent with this study, we found that rats in the IS group presented more immobility and less movement, similar to the avoidance of routine activity in migraineurs during attacks [30]. In clinical trials, topiramate has been proved effective for migraine prevention [22]. Also, topiramate can attenuate both acute and chronic hyperalgesia in an NTG-induced migraine model [31]. Our results showed that rats treated with topiramate showed significant reductions in the IS-induced allodynia and nociception-related behaviors.

Recent studies have demonstrated that bidirectional interactions of the gut-brain axis are involved in the pathophysiology of neuropsychiatric disorders as well as gastrointestinal diseases [32]. Migraineurs often suffer from gastrointestinal comorbidities, including *Helicobacter pylori* infection, IBS, IBD and celiac disease [3]. Patients with IBS have a higher prevalence odd of migraine, while migraineurs with a long headache history and high headache frequency have a higher chance of being diagnosed with IBS [33, 34]. The roles of intestinal microbiota and gut-brain axis may provide plausible explanations for migraine and its gastrointestinal comorbidities [5]. Host-microbiota interactions participate in various physiological and pathological events, including host homeostasis, energy metabolism, cognition, behavior and nociception [35, 36]. In a study of 108 elderly women, discrepancy was found in the gut microbiota composition between migraineurs and healthy controls [37]. But in humans, daily diet and lifestyle also have an impact on the gut microbiota, indicating the complexity and diversity of microbial profiles [38]. Here, we found microbial composition alteration in the IS group compared with the VEH group. However, in the IS-TO group, prophylactic treatment with topiramate could prohibit IS-induced alteration in the gut microbiota. These results led us to hypothesize that migraine-related cephalic allodynia induced alteration in the gut microbiota composition.

Although little research demonstrated the association between microbial dysbiosis and migraine, some studies have reported that migraineurs benefit from a 10–12 week probiotic mixture containing strains of *Lactobacillus* and *Bifidobacterium* [39, 40]. In our research, the relative abundances of both the *Lactobacillaceae* family and *Lactobacillus* genus were significantly decreased in the IS group compared with the VEH group, indicating reduced probiotic strains and intestinal dysbiosis. However, more research is needed to confirm the association between the reduction of beneficial bacteria and the probiotic supplement treatment in migraine. Among the five biomarker genera, four were increased in the IS group, including *Colidextribacter*, *Lachnoclostridium*, *Monoglobus* and *Anaerovorax*, while *Lactobacillus* was not. Previous studies have shown that *Colidextribacter* is positively correlated with antidepressive-like behavior and that *Monoglobus* may participate in pectin degradation [41, 42]. Both *Lachnoclostridium* and *Anaerovorax* are butyrate-producing genera [43, 44]. The highest LDA score as well as MDA score for *Lactobacillus* indicated that reduced *Lactobacillus* was the most prominent biomarker genus in the IS-induced alteration of microbiota composition.

For microbial functional predictions, we found that in comparison with the VEH group, the butanoate, propanoate, and tryptophan metabolic pathways were enhanced in the IS group. Microbiota-related products in these pathways, including SCFAs and tryptophan metabolites, have been reported to participate in pain modulation [12, 15].

Short-chain fatty acids, mainly acetate, propionate and butyrate, are fermentation products of undigested carbohydrates produced by intestinal microbiota. Propionate can affect fatty acid content as well as food intake, and also inhibit inflammation in the gastrointestinal tract [45]. Kukkar et al. reported that a 14-day administration of butyrate attenuates neuropathic pain in a chronic constriction injury rat model [12]. Recent research has shown that both propionate and butyrate can reduce pain attacks in an NTG-induced migraine model [17]. The anti-inflammatory effects of SCFAs can down-regulate the levels of proinflammatory mediators in the trigeminal nucleus, including cyclooxygenase-2 (COX-2) and inducible nitric oxide synthase (iNOS) [17]. Thus, SCFAs are likely to be involved in alleviating NTG-induced neuroinflammatory response and inhibiting nociceptive transmission. Although enhanced butanoate and propanoate metabolic pathways were predicted in the IS group, we failed to detect any significantly different SCFAs in either feces or sera.

Tryptophan is an essential amino acid for humans, and its metabolites are crucial in host-microbiota interactions. On one hand, the gut microbiota could directly utilize tryptophan, reducing its availability to the host [46]. On the other hand, microbiota-related tryptophan metabolites can enter the circulation, distributing to target sites with diverse biological activities [46]. The migraineurs suffer from a low central 5-HT disposition associated with an increase in 5-HT release during attacks, indicating disturbances of tryptophan metabolism in migraine [47]. Kiss et al. reported that cortical KYNA level is augmented in a migraine model of cortical spreading depression, seeming to decrease the sensitivity in the cerebral cortex [48]. Additionally, LC–MS/MS analysis revealed that serous KYNA level is significantly decreased in chronic migraine patients compared with healthy controls [49]. However, in our study, neither serotonin nor kynurenine-related metabolites were found significantly different between the IS and VEH groups. A possible explanation could be that 7 times of IS infusion were not enough to mimic an abnormal serotonin or kynurenine metabolic state in rats. Interestingly, we found that the indole-related derivatives IAM and IEt were significantly increased in feces of the IS group compared with the VEH group. These results revealed that elevated IAM and IEt in feces could be the indicators in the IS-induced alteration of microbial metabolism.

In our previous study, we have found that eradication of gut microbiota leads to increased basal mechanical sensitivity and hyporesponsiveness to NTG administration in mice, which could be reversed by restoring the gut microbiota [16]. However, Tang et al. reported that gut microbiota deprivation dramatically prolongs NTG-induced migraine-like orofacial pain in mice [50]. Despite these results are not entirely consistent, the gut microbiota seems to be associated with pain modulation in rodent models of migraine. In addition, these two studies have demonstrated that upregulation of tumor necrosis factor alpha (TNF-*α*), especially in the trigeminal nucleus caudalis, influences NTG-induced acute hyperalgesia [16, 50]. As an important proinflammatory cytokine, TNF-*α* is involved in the innate immune response [51]. In pathological conditions, microglia can release large amounts of TNF-*α*, which contributes to neuroinflammatory response [52]. Erny et al. discovered that deprivation of gut microbiota causes microglia function defects in mice, affecting the release of TNF-*α* [53]. This may provide a plausible explanation for our previous observations that NTG could not induce elevation of TNF-*α* in both germ-free and antibiotic-treated mice. Several studies have revealed that microbiota-derived SCFAs play important roles in modulating microglia homeostasis as well as inhibiting neuroinflammatory response [17, 53, 54]. Wen et al. reported that *Gastrodia elata* Blume and *Uncaria rhynchophylla*, a Chinese medicine pair, can affect the microbial composition and amino acid metabolism, and alleviate NTG-induced thermal allodynia in rats [55]. These findings have shown potential therapeutic targets for migraine by regulating the gut microbiota and its metabolites.

In this study, we mainly investigated that IS-induced cephalic allodynia caused the alteration of gut microbiota composition. Although the exact mechanism is still unknown, there are several hypotheses to explain how the CNS modulates gut microbiota. On one hand, the CNS can influence intestinal microbiota by sending efferent signals from the sympathetic and parasympathetic systems [9]. On the other hand, physical or psychological stress factors can act on the hypothalamic-pituitary-adrenal (HPA) axis and induce cortisol secretion, causing alterations of the microbiota profile and intestinal permeability [56]. It is worth noting that stress factors can induce microbial composition alteration in a short time. In one animal model of depression, the mice are exposed to a different CD1 aggressor mouse for 10 min on 10 consecutive days, and then alteration in the gut microbiota is found [57]. In another animal model of depression, the gut microbiota composition alters when the rats have been subjected to inescapable electric foot shocks for two days (30 times per day) [58]. In our research, we found the alteration of gut microbial composition after 7 times of IS dural infusion. However, the underlying mechanisms through which cephalic allodynia influences the gut microbiota have not yet been defined and future research is needed.

Based on the above, we speculated that when the host confronts with stress factors or environmental changes, such as migraine attacks, the adaptive alterations occur in the gut microbiota. To some degree, these alterations might be beneficial for the host adapting itself to migraine. As migraine progresses, gut microbial dysbiosis and altered microbiota-related metabolites emerge. Reversely, these changes might affect the progression of migraine through the gut-brain axis. Therefore, the bidirectional host-microbiota crosstalk might be involved in the migraine pathophysiology.

It should be noted that migraine is highly prevalent in women, with a female-to-male ratio of 3:1 [1]. There is a strong relationship between migraine and female hormones, and the fluctuations of estrogen have an impact on intensity and duration of the headaches in migraineurs [59, 60]. Additionally, accumulating evidence suggests that there also exists bidirectional crosstalk between estrogen and the intestinal microbiota [61, 62]. Considering the effects of female hormones on pain processing and gut microbiota, only male rats were used in our study. Whether cephalic allodynia would influence the gut microbiota in female rats needs to be further explored. There are still some limitations in the present study. This animal model induced by repeated dural IS infusions may partially simulate the pathophysiology of migraine. Due to the limited times of IS administration, the microbial composition alteration might be a stress-induced phenomenon. Hence, it is inconclusive whether recurrent headaches would induce alterations of the microbiota profile and microbial metabolic pathways in migraineurs. Clinical study is needed to investigate the mechanism of host-microbiota interactions in the future.

## Conclusions

In conclusion, our study indicates that inflammatory dural stimulation-induced cephalic allodynia causes alterations of the microbiota profile and microbial metabolic pathways. Decreased *Lactobacillus* is the most prominent biomarker genus, while increased IAM and IEt in feces are the indicator metabolites. Further studies focusing on the mechanism of bidirectional interactions are needed to develop novel approaches for the prevention or treatment of migraine.

## Data Availability

The datasets generated or analyzed during the current study are available from the corresponding author on reasonable request.

## Abbreviations

IBS: irritable bowel syndrome
IBD: inflammatory bowel disease
CNS: central nervous system
SCFAs: short-chain fatty acids
5-HT: 5-hydroxytryptamine (serotonin)
KYNA: kynurenic acid
NTG: nitroglycerin
IS: inflammatory soup
GC–MS/MS: gas chromatography-tandem mass spectrometry
LC–MS/MS: liquid chromatography-tandem mass spectrometry
SPF: specific pathogen-free
DDW: double distilled water
PBS: phosphate buffered saline
PCR: polymerase chain reaction
OTU: operational taxonomic units
MRM: multiple reaction monitoring
PCoA: principal coordinates analysis
PERMANOVA: permutational multivariate analysis of variance
ANOSIM: analysis of similarities
LDA: linear discriminant analysis
LEfSe: linear discriminant analysis effect size
RF: random forest
MDA: mean decrease accuracy
KEGG: Kyoto Encyclopedia of Genes and Genomes
FDR: false discovery rate
ANOVA: analysis of variance
LSD: Fisher’s least significant difference
IAM: indole-3-acetamide
IEt: tryptophol (indoleethanol)
COX-2: cyclooxygenase-2
iNOS: inducible nitric oxide synthase
TNF-*α*: tumor necrosis factor alpha
HPA: hypothalamic-pituitary-adrenal
